# Employee perception of precision technology use at the dairy farm

**DOI:** 10.1093/tas/txae036

**Published:** 2024-03-22

**Authors:** Natalia Herrera, Juan Vélez, Timothy Holt, Pablo Pinedo

**Affiliations:** Department of Animal Sciences, Colorado State University, Fort Collins, CO 80523, USA; Aurora Organic Farms, Platteville, CO 80651, USA; Department of Clinical Science, Colorado State University, Fort Collins, CO 80523, USA; Department of Animal Sciences, Colorado State University, Fort Collins, CO 80523, USA

**Keywords:** employee, perception, precision dairy, survey, technology

## Abstract

The adoption of precision technologies on dairy farms has increased significantly in recent decades, leading to the challenge of providing employees with resources to maximize the efficient use of these tools. The objective of this study was to explore how dairy farm employees perceive the available precision technologies and to identify possible challenges they face when adapting to their use at the farm. An online survey consisting of four sections (employee demographics, precision technologies in use, perception of these technologies, and opportunities for adapting to technology use) was completed from September to December 2022 by 266 farm employees from three dairies operated under similar management. Most of the respondents were identified as male (72.2%), Hispanic or Latino (92.5%), aged between 21 and 30 (39.1%) or 31 and 40 yr (36.8%), with a bachelor’s degree (34.6%) or completion of middle school (29.3%) and having basic or no English proficiency (74%). Overall, the respondents indicated being comfortable (95.6%) with and understanding (91.8%) the technology they use. Employees recognized precision technology as a tool that helps them to be more efficient (93.7%), identifying the technologies’ benefits (92.1%). However, challenges for adapting to these technologies included personal limitations, such as not knowing the language of the technology (31%), visual impairments (24%), light sensitivity (14%), and not being able to read (7%). Environmental limitations were also recognized and included cold weather (64.3%), wind (46%), and surroundings that were too dark (31%) or too bright (21%). Significant associations between perception of the technology and age, level of education, and English proficiency were identified. Respondents indicated their desire to learn more about precision technologies implemented at work, which could eventually lead to improved efficiency at the dairy operation through innovations in the way users interact with these technologies, increasing employees’ motivation. This study provides insights that could assist the dairy industry in addressing challenges and enhancing opportunities for a more efficient use of precision technologies at dairy farms.

## Teaser

Most dairy employees in the current study had a positive perception of precision dairy technologies and recognized the benefits provided by their use in their daily work routine.

Challenges associated with language and environmental conditions were identified.

The current findings highlight some critical points affecting the success of the implementation of precision technologies from the perspective of farm employees.

## INTRODUCTION

Over the past decades, agricultural operations have shown a significant surge in the implementation of technology (El-Osta et al., 2000; Fuentes et al., 2022). The dairy industry has also followed this trend, integrating precision dairy (**PD**) technologies in general management, feeding, animal health monitoring, and data integration software (IFCN, 2022; Jiang et al., 2023). As presented by Eastwood et al. (2012), the main goal of PD is to optimize economic, social, and environmental farm performance by the use of information and communication technologies. In practical terms, PD integrates the use of sensor technology, algorithms, interfaces, and applications in animal husbandry (Kleen and Guatteo, 2023). Moreover, these technologies can be affixed to the animal (i.e., ear tags, collars, pedometers), incorporated into the milking system, stand-alone, or part of the herd management software (Borchers and Bewley, 2015; Bewley et al., 2017).

The adoption of PD technologies and automation has accompanied and supported the dairy industry’s consolidation toward larger operations with increased numbers of high-producing cows (MacDonald et al., 2020). The perceived benefits of PD implementation in large dairies include improving productivity and product quality, lowering per-unit costs (El-Osta and Morehart, 2000), addressing labor shortages (Lovarelli et al., 2019; Charlton and Kostandini, 2021), increasing labor efficiency, and improving decision-making processes (Hogan et al., 2022). Another perceived benefit of PD technologies is the ability to direct the focus of animal welfare and health monitoring strategies on both the individual and the group (Bewley, 2010; Jago et al., 2013; Barkema et al., 2015; Tullo et al., 2019). Some examples of PD technologies reducing pressures on the labor force include automatic teat cup detachers, sorting gates, calf feeders, and post-milking disinfection (Edwards et al., 2015, 2020). On the other hand, PD technologies aimed at increasing production efficiency mostly rely on data capture and cow monitoring and include automatic estrous detection, inline milk meters, electronic cow identification systems, and herd management software (Gargiulo et al., 2018).

Notably, despite the widespread availability and potential beneficial impacts of PD technologies, their adoption at farms has been slower than expected (Gelb et al., 2001; Bewley 2010). In a study by Bewley and Russell (2010), the most common reasons for the modest adoption of PD technologies were unfamiliarity with the technologies, undesirable cost–benefit ratios, and the excess of information without knowing what to do with it. These were followed by a lack of time spent receiving training, a lack of perceived economic value, and the idea of high complexity. Moreover, Bewley et al. (2010) and Borchers and Bewley (2015) identified uncertainty about market conditions, technology performance, and the impact of the technology on the management of the farm as perceived limitations for investment in PD technologies.

Although multiple surveys investigating PD technology adoption have been conducted consulting dairy farmers in different countries (Khanal et al., 2010; Borchers and Bewley, 2015; Edwards et al., 2015; Kleen and Guatteo, 2023), less is known about the impact of hired labor on the success of on-farm implementation of precision technologies. As pointed out by Shang et al. (2021), the success of PD adoption relies on multiple factors that are variable among farms, including the compatibility of new farming technologies with existing machinery and the available telecommunication infrastructure. Features of farm operators such as level of education and computer literacy are also considered relevant. In agreement, in a study by Eastwood (2008), the lack of skilled labor was identified as a key limitation for investment in PD technologies, while Jago et al. (2013) indicated that farmers recognized the accompanying challenge of human resource management, mentioning the difficulty of finding suitably skilled workers to operate in these systems. Moreover, Eleveld et al. (1992) demonstrated that PD technology adoption improves when the technology fits within the daily work patterns of the personnel and when farm operations have more specialized labor. Importantly, as foreign-born migrant workers represent a significant proportion of the labor force in U.S. agriculture (NORA, 2018), language barriers are potential challenges for the appropriate implementation of PD technologies.

In consequence, a better understanding of the needs and barriers that farm workers face when working with PD technologies would be informative for farm management teams, manufacturers, and the dairy industry in general. Therefore, the objective of this study was to explore how dairy farm employees perceive the available precision technologies and to identify possible challenges they face when adapting to their use at the farm.

## MATERIALS AND METHODS

### Study Design and Study Population

This study was approved by the Colorado State University Institutional Review Board (IRB Protocol #3459) and the researchers involved in the development of the online survey obtained CITI Human Research Behavior certification. An online survey was developed by the researchers and made available from September to December 2022 to dairy employees in three dairies operated under the same ownership and general management. The surveyed farms were selected through convenience sampling, considering the size of the operations, the number of employees, and the use of PD technologies. The survey included four sections (employee demographics, precision technologies in use, perception of these technologies, and opportunities for adapting to technology use). The survey was distributed through SurveyMonkey (SurveyMonkey Inc., Palo Alto, CA) and available in English and Spanish. To protect participant anonymity, no personal identifiers were used and the online survey information was shared among the private social media of the company. A QR code flier was provided, which linked to the online survey that could be completed by using the employees’ smartphones. To avoid more than one response to the survey by the same employee, the multiple responses feature of the survey was turned off.

To be eligible, participants had to work at the dairy and had to be over 21 yr old. Moreover, one investigator offered sessions scheduled on-site for 3 mo (10 sessions in the Colorado locations and 3 sessions in the Texas farm, covering 6 to 8 h/d) to assist if participants needed help completing the online survey. The help sessions were limited to delivering the study consent, explaining the research purpose, and reading the survey in case the participant required it. The survey was voluntary and completed by employees at their work location, including animal-related workers (animal health, animal caretakers, animal feeding, and milkers), agricultural resources employees (pasture management, machinery, and equipment specialists), and management team members in the dairy, such as unit managers and area supervisors (dairy unit, maternity, dry cows, calf yard, maintenance, etc.). Eleven participants’ responses from temporary employees who had just arrived in the operations were deleted from the study.

### Management, Operation Size, and Technology

Of the three farms surveyed, two were located in Northern Colorado (CO1 and CO2) and one in Northern Texas (NT). All farms were managed under the same human resources guidelines and adopted similar PD technologies. However, there were a few differences in the inventory technology among farms, either regarding the brand or quantity of technologies adopted. The surveyed dairies housed 15,300 (CO1), 13,600 (TX), and 1,500 (CO2) animals, including lactating and dry cows, heifers, and calves. Cows in the smallest CO dairy (CO2) were housed in dry lots, with access to shade and shelter that included straw bedding. Cows in the largest CO farm and in the TX location were maintained in freestall barns, provided with sand bedding, headlocks, and access to an outdoor dry lot and to ad libitum water. In all the farms, cows were fed a total mixed ration (TMR) twice a day to meet or exceed the nutritional requirements for a lactating Holstein cow producing 30 kg/d of milk with 3.5% fat and 3.1% true protein. During the grazing season (April to September), cows had access to pasture and grazing provided at least 30% of the dry matter intake I of the total ration. Cows in CO2 were milked in an automatic milking system (Lely Industries NV, the Netherlands) and in a parallel milking parlor (DeLaval International AB, Sweden). Milking parlors in CO1 included rotary parlors (DeLaval) and a parallel milking parlor (DeLaval). Cows in the TX location were milked in parallel milking parlors (DeLaval).

### General Procedures

**Figure 1.**
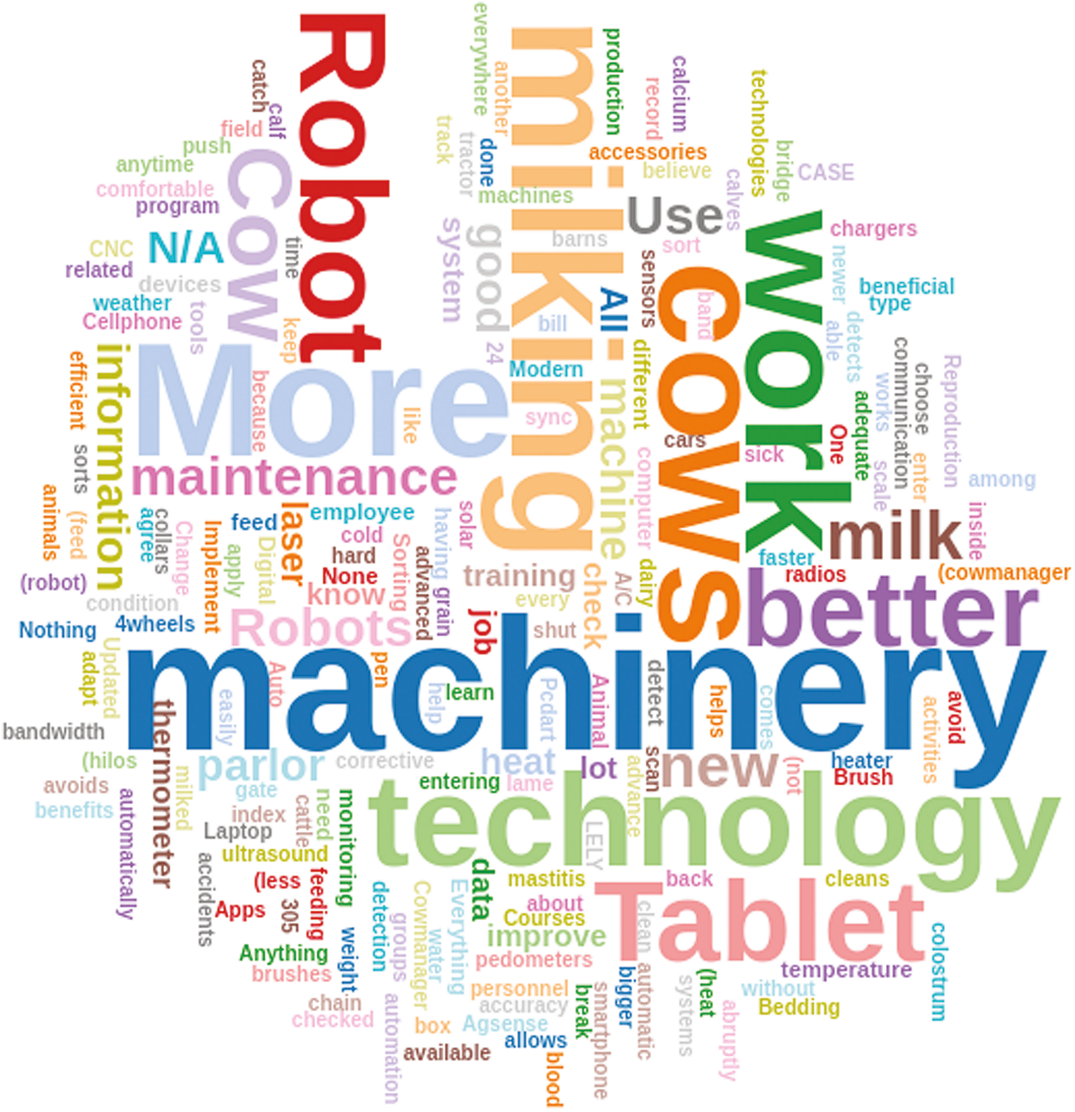
Word Cloud responses from employees (*n* = 266) in three dairy farms within the same operation to the opportunities to adapt question: “Which technology do you think will be beneficial for your work?” The chi-square test of independence indicated significant differences among word frequencies (*P* < 0.01).

**Figure 2.**
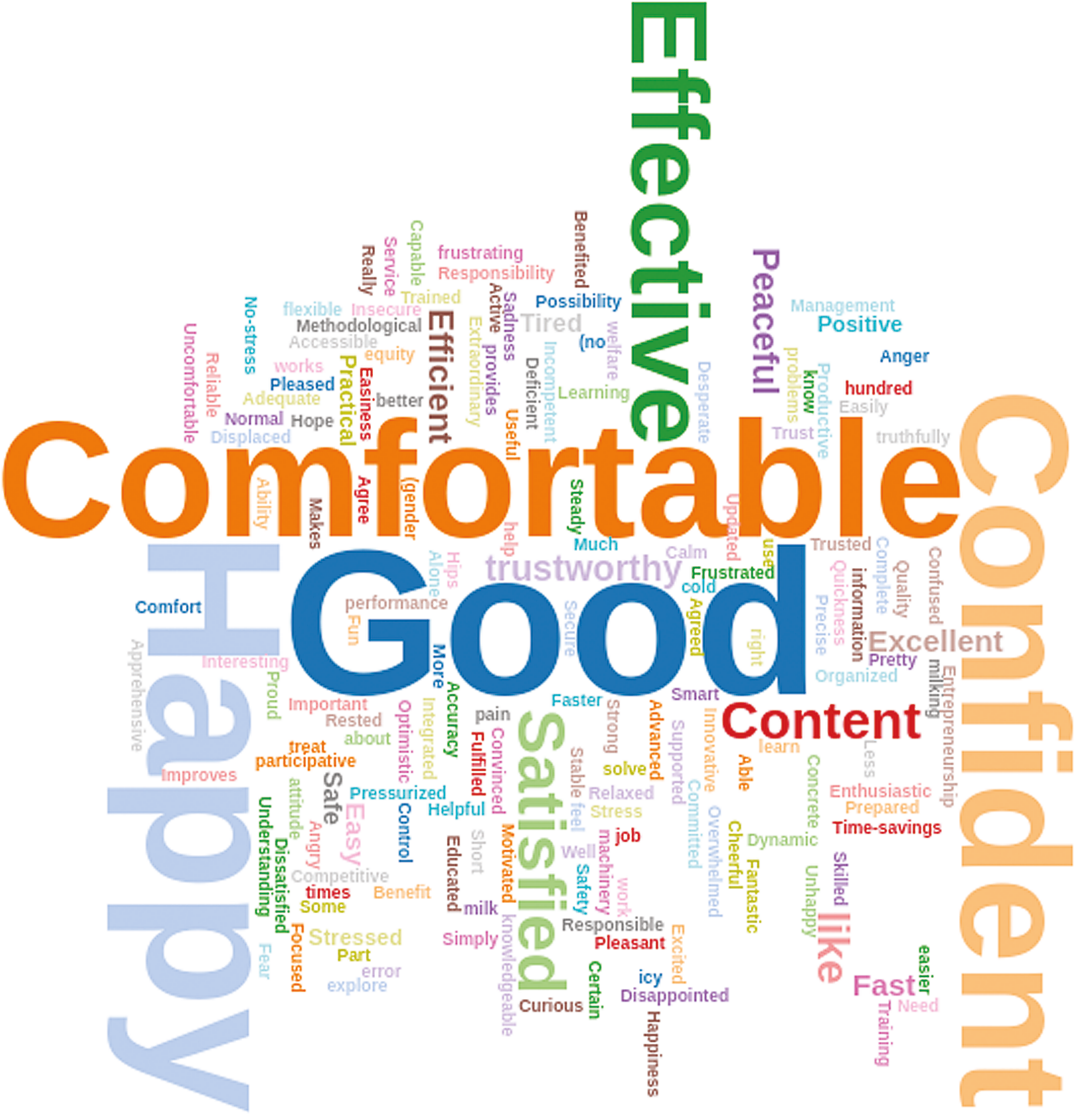
Word Cloud responses from employees (*n* = 266) in three dairy farms within the same operation to the opportunities to adapt the question: “In three words, describe how the technology implemented at work makes you feel?” The chi-square test of independence indicated significant differences among word frequencies (*P* < 0.01).

**Figure 3.**
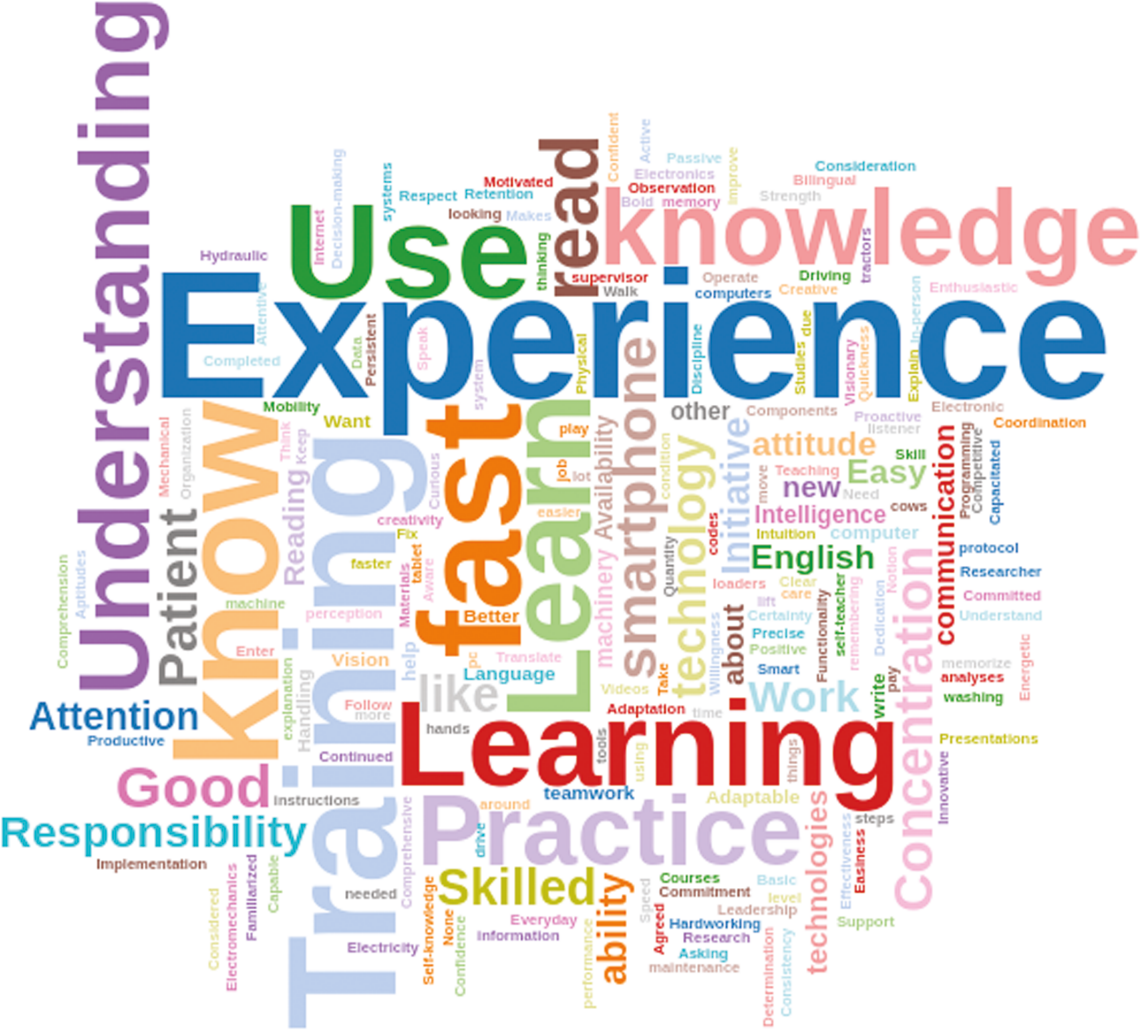
Word Cloud responses from employees (*n* = 266) in three dairy farms within the same operation to the opportunities to adapt the question: “Mention three skills you consider that help you understand the technology at work.” The chi-square test of independence indicated significant differences among word frequencies (*P* < 0.01).

Respondents were asked to voluntarily complete the online survey with four main sections (Supplementary Appendix 1). The first section was a demographic and general questionnaire that consisted of questions such as respondents’ age, gender, ethnicity, level of education, primary language, perceived English proficiency, frequency of personal smartphone use, and department within the dairy operation. Secondly, respondents were asked to complete a technology-related questionnaire inquiring which PD technologies they use in their work at the dairy farm. The beginning of the technology questionnaire offered the following definition: “precision dairy farming is defined as the use of information and communication technologies to improve animal management.” The third section was focused on the employee perception of PD technologies and used a 5-point Likert scale in which respondents indicated their agreement (5—strongly agree, 4—agree, 3—neutral, 2—disagree, 1—strongly disagree, 0—does not apply). Statements in this section were focused on the specific PD technologies used at work. The final section related to opportunities for adapting to the technologies, with multiple choice and open-ended questions to be represented in word clouds that intended to identify challenges and opportunities for a more efficient use of PD technologies. No filtering of terms that may not be related to precision technologies was attempted in the visual representation of data. Open-ended questions that were responded in Spanish were translated by one of the authors (N.H.) for reporting in Figs. 1–3.

### Statistical Analysis

Data from the survey were imported into Microsoft Office Excel 2011 (Microsoft, Redmond, WA, USA), while Microsoft Office Word 2011 (Microsoft) was used to organize qualitative data (open-ended questions). The analyses were conducted using SAS Version 9.4 (SAS Institute Inc., Cary, NC, USA). Frequency distributions were calculated to describe the responses from the demographic, use of technology, and perception of technology questionnaires (PROC FREQ). An initial screening to assess potential associations between selected questions in the demographic section (age, gender, level of education, and English proficiency) and each of the 16 questions in the perception of PD technologies section was completed by using the chi-square test of independency (PROC FREQ). Subsequently, the responses evidencing associations (*P* < 0.05) were analyzed by cumulative logit models for ordinal responses (PROC GLIMMIX; link = cumlogit) to evaluate the magnitude and direction of these associations (Agresti, 1996). For these analyses, the responses on a Likert scale were transformed to values from 1 (strongly disagree) to 5 (strongly agree).

A chi-square test of independence was used to establish differences in the word frequencies presented in the word clouds. For all the association tests, significant predictors were determined at *P*-value < 0.05.

## RESULTS

### Demographics and PD Technology Use

A general description of the participant population from the demographic section of the questionnaire is presented in Table 1. In total, the survey was completed by 266 out of 355 farm employees in the three targeted farms. Only 10 employees submitted the survey presented in English. Overall, 72.2% of participants identified as male. The higher proportion for age was between 21 and 30 yr (39.1%), followed by employees between 31 and 40 yr (36.8%). Most employees identified themselves as Hispanic or Latino (92.5%), followed by respondents who identified as White or Caucasian (4.9%). The majority of respondents completed a bachelor’s degree (34.6%), followed by completion of middle school (29.3%) and elementary school (10.2%). Almost half (42.1%) of the participants indicated no English proficiency and 31.9% considered themselves to have basic English level. Regarding the distribution of participants by departments, depending on their task at the dairy, most respondents were involved in milking (30.8%), animal care (23.3%), or operations and maintenance (16.9%). Most of the responders indicated that they use a personal smartphone daily (93.2%).

**Table 1.** Responses from employees (*n* = 266) in three dairy farms within the same operation to the demographic questionnaire including gender, age, ethnicity, level of education, and English proficiency

Demographic variables^1^	Response (*n*)^2^	Response, %
Gender		
Female	63	23.9
Male	192	72.2
Prefer not to answer	8	3.01
Non-binary	3	1.1
Age		
21 to 30 yr	104	39.1
31 to 40 yr	98	36.8
41 to 50 yr	36	13.5
Over 50 yr	28	10.5
Ethnicity		
Asian or Pacific Islander	3	1.1
Hispanic or Latino	246	92.5
Native American or Alaskan Native	2	0.7
White or Caucasian	13	4.9
Ethnicity not described	2	0.7
Level of education		
None	24	9.02
Elementary school	27	10.2
Middle school	78	29.3
High school	23	8.6
Technician	9	3.4
Bachelor	92	34.6
Master’s degree or above	9	3.4
Other	4	1.5
Primary language		
English	16	6.02
Spanish	248	93.2
Other	2	0.7
Level of English (perceived)		
None	112	42.1
Basic	85	31.9
Intermediate	39	14.6
Advanced	17	6.4
Native	13	4.9
Dairy operation department		
Animal caring	62	23.3
Animal reproduction	23	8.6
Calf/ heifer raising	25	9.4
Farm office and administration	15	5.6
Feeder	23	8.6
Milking related	82	30.8
Operator/maintenance	45	16.9
Supervisor/manager	17	6.4

^1^Responses were attained anonymously from the online survey.

^2^Frequency response over the total sample (*n* = 266), except for “Dairy operation department” where more than one primary department could be indicated. Self-reported by dairy employee.

A summary inventory of PD technologies by the surveyed farm is shown in Table 2. Regarding the use of PD technology in their work, 88.4% of respondents recognized using technology in their daily routines, with 86% using technology every working day, and 5% using it only two to three times per week. The greatest use of technology was associated with cow milking and with monitoring animal health.

**Table 2.** Dairy farm inventory of technology per farm (three farms) by area of application

Technology resource (manufacturer)	Farm^1^
Animal identification and monitoring	
Collars (Lely)	CO2
CowAlert (Peacock Technology)	CO1, NT
DS i-QUBE pedometers (Peacock Technology)	CO1, NT
CowManager Ear Sensor (CowManager)	CO2
One Wand (Allflex)	All
DelPro Animal Tracking (DeLaval)	All
Sorting gate (DeLaval)	All
Milking	
Milking parlor (Rotary, DeLaval)	CO1
Automatic Milking System (Lely)	CO2
Parallel milking parlor	CO1, CO2, TX
Data management	
PCDART Software (Pocket and PC; DRMS)	All
PocketDairy (DRMS)	All
Feeding	
Milk pasteurizer (Perfect Udder, Dairy Tech Inc.)	All
Calf feeder (Lely)	CO2
Automatic feed pusher system (GEA)	All
TMR Tracker Software (Topcon)	All
Tractor/loader (Case/John Deere/Kubota machinery)	All
Other	
Swinging cow brush (DeLaval)	All
Pivots water management (AgSense Technology)	All
Ultrasound for pregnancy diagnosis	All

^1^The participant dairy farms were categorized as CO1—Northern Colorado 1, CO2—Northern Colorado 2, and TX—Northern Texas.

### Perception and Understanding of PD Technologies

Responses on the perception of PD technologies are summarized in Table 3. Most responders agreed (combining Likert scores agree and strongly agree) that they feel comfortable using PD technology in their daily routine (95.6%) and when explaining to a colleague how the technology works (90.1%). Most agreed on understanding the technology (91.8%) and its benefits (92.1%), while recognizing that the technology helps them to be more efficient (93.7%). A similar proportion considered that the data provided by the technology were reliable (84.1%) and felt confident with the information obtained from that source (89.0%).

**Table 3.** Likert responses frequency from employees (*n* = 266) in three dairy farms within the same operation to the perception and understanding of technologies questionnaire

Perception and understanding of technology question^1^	5		4		3		2		1	
	*n*, %		*n*, %		*n*, %		*n*, %		*n*, %	
1. I feel comfortable using technology in my daily routine.	159	(61.8)	87	(33.8)	5	(1.9)	3	(1.20)	3	(1.20)
2. I understand how technology works.	136	(53.1)	99	(38.7)	17	(6.6)	3	(1.20)	1	(0.40)
3. I can easily set up or back up the technology if it is not working.	46	(19.4)	76	(32.1)	60	(25.3)	32	(13.5)	23	(9.70)
4. I feel comfortable with the language in the technology I am using.	116	(45.6)	94	(37.0)	30	(11.8)	12	(4.70)	2	(0.80)
5. I feel comfortable explaining to my colleague how to use the system.	125	(48.4)	108	(41.7)	21	(8.1)	3	(1.20)	1	(0.40)
6. Technology helps me to be more efficient in my daily operations.	168	(64.8)	75	(28.9)	13	(5.02)	2	(0.70)	1	(0.40)
7. I understand the benefits of the technology that I am using.	149	(58.2)	87	(33.9)	17	(6.6)	2	(0.70)	1	(0.40)
8. I recognize that the information I enter in the system, someone else is using it.	114	(50.0)	82	(35.9)	26	(11.4)	3	(1.30)	3	(1.30)
9. I feel confident using the information from the technology that I am using.	129	(50.8)	97	(38.2)	22	(8.60)	4	(1.50)	2	(0.80)
10. I use technology or the information from the technology to make decisions in my job.	111	(46.1)	91	(37.7)	29	(12.0)	7	(2.90)	3	(1.20)
11. The dairy provides me with all the resources I need to use the technology.	114	(44.2)	93	(36.1)	39	(15.1)	10	(3.80)	2	(0.80)
12. The data resulting from monitoring systems are reliable.	99	(40.4)	107	(43.7)	33	(13.5)	5	(2.04)	1	(0.40)
13. I feel comfortable using new technology when implemented at my work.	120	(46.3)	114	(44.0)	18	(6.9)	6	(2.30)	1	(0.40)
14. I get training when there is new technology in my work.	114	(44.0)	102	(39.4)	26	(10.0)	13	(5.02)	4	(1.50)
15. I have a positive attitude when adapting to new technology at my work.	154	(58.8)	88	(33.6)	20	(7.6)	0	—	0	—
16. I would like to learn more about using technologies at work.	182	(69.2)	68	(25.9)	10	(3.8)	1	(0.4)	2	(0.7)

^1^Likert scale questions from survey: scale 5 to 0; 5—strongly agree response, 4—agree, 3— neutral, 2—disagree, and 1—strongly disagree response; score 0—does not apply responses were excluded from the frequency analyses.

Regarding the resources that the dairy provides for the use of PD technologies, 83.4% of employees indicated that they received training when using a new technology, 90.3% felt comfortable when a new technology was implemented, and 92.4% had a positive attitude when adapting to new technologies. Nonetheless, the responses about the level of comfort to setting up or backing up a technology when it was not working were divided into neutral (25.3%) and disagreement (23.2%, combining Likert scores of disagree and strongly disagree). Overall, 95.1% of employees agreed on their desire to learn more about new PD technologies at the farm.

### Opportunities to Adapt to PD Technologies

In the adapting to PD technology use section of the questionnaire, responders indicated that the main personal limitation in the use of this technology was not knowing the language of the technology (31%), followed by visual impairments (24%), light sensitivity (14%), and not being able to read (7%). Respondents agreed with cold weather being the most significant environmental limitation for technology use (64.3%), followed by wind (46%), when it is too dark (31%), when it is too bright (21%), hot weather (18%), rain (7%), and snow (5%). Responses to the open-ended question “Which technology do you think will be beneficial for your work?” are represented in a word cloud, illustrated in Fig. 1. The most represented responses were for “machinery” and “milking.” Although word clouds provide a visual representation of word frequencies (Hearst et al., 2019), some limitations include the potential use of broad terms, such as “machinery,” “more,” or “cows,” that were presented in high frequency in the current survey.

Employees indicated that they would like to have more robots (such as robots for milking cows, robots that help us to push the cows to the milking parlor, robots that sort the cows themselves, robots that detect lame cows or cows with diseases, robot to apply iodine, and more robot barns). This was followed by responses indicating maintenance tools and machinery (“program that monitors the maintenance of vehicles,” “good maintenance accessories on hand,” “tools in general for maintenance,” and “more courses or videos to learn more in the maintenance field”). Also, live uploading of data from tablets was mentioned by the dairy employees (“internet in the workplace to sync the tablet,” “all information uploaded automatically without the need to sync tablets,” and “implement a technology that could connect to the tablet and sync immediately, especially for the weekends”).

Responses to the request to list three emotions or feelings for “How does the technology makes you feel?” are likewise represented in a word cloud, illustrated in Fig. 2. Overall, feelings and emotions toward technology were positive, with the greatest frequencies for “happy,” “good,” “comfortable,” and “confident.” A few negative emotions, such as feeling “stressed,” “uncomfortable,” “frustrated,” and “tired,” were also presented. Finally, when requested to list three skills considered helpful for understanding PD technologies, “experience,” “training,” “use,” and “learning” were the most common responses (Fig. 3).

### Association Tests

Results from the screening analyses for testing potential associations between participant perception of PD technology and gender, age, level of education, and English proficiency are summarized in Table 4. Overall, gender was not associated with the responses to any of the questions presented in the perception section of the questionnaire. Age was associated with the ability to set up or back up a technology that was not working, with the use of information from the technology to make decisions, and with being comfortable using a new technology implemented at work. Level of education was associated with the ability to set up or back up a technology, with perceiving that technology improves efficiency, with understanding the benefits of precision technology, with recognizing that somebody else uses the information that has been generated by that technology, and with the desire of learning about the use of technology. Finally, English proficiency was significantly associated with the ability to set up or back up a technology that was not working, with understanding the benefits of precision technology, with the use of information from the technology to make decisions, with receiving training when exposed to a new technology, and with a positive attitude when adopting a new technology (Table 4).

**Table 4.** Resulting *P*-values from the chi-square test of association testing associations between the perception and understanding of technologies questionnaire and gender, age, level of education, and English proficiency

Outcome: perception and understanding of technology question	*P-*value			
	Gender	Age	Level of education	English proficiency
1. I feel comfortable using technology in my daily routine	0.88	0.87	0.05	0.84
2. I understand how technology works	0.84	0.53	0.37	0.50
3. I can easily set up or back up the technology if it is not working	0.20	0.03	0.00	0.03
4. I feel comfortable with the language in the technology I am using	0.90	0.34	0.60	0.14
5. I feel comfortable explaining to my colleague how to use the system	0.80	0.83	0.35	0.97
6. Technology helps me to be more efficient in my daily operations	0.63	0.49	0.04	0.05
7. I understand the benefits of the technology that I am using	0.79	0.39	0.02	0.04
8. I recognize that the information I enter in the system, someone else is using it	0.99	0.32	0.01	0.23
9. I feel confident using the information from the technology that I am using	0.95	0.70	0.29	0.99
10. I use technology or the information from the technology to make decisions in my job	0.97	0.05	0.21	0.03
11. The dairy provides me with all the resources I need to use the technology	0.79	0.74	0.05	0.32
12. The data resulting from monitoring systems are reliable	0.92	0.92	0.93	0.92
13. I feel comfortable using new technology when implemented at my work	0.89	0.03	0.57	0.89
14. I get training when there is new technology in my work	0.94	0.84	0.23	<0.01
15. I have a positive attitude when adapting to new technology at my work	0.88	0.18	0.10	0.02
16. I would like to learn more about using technologies at work	0.36	0.91	<0.01	0.10

Cumulative logit models for ordinal responses were used to examine the size and direction of the associations between the perception of PD technologies section of the questionnaire and the demographic variables that were identified as significant through a chi-square test (age, level of education, and English proficiency). Age was found to be a significant factor associated with one of the variables tested. In specific, the odds of agreeing that the use of PD technology improves decision-making in their job multiplied by 0.34 (95% CI: 0.14 to 0.39) with participants between 21 and 30 yr old compared to employees more than 50 yr old.

Regarding the level of education, when using “no formal education completed” as a reference category, the odds of understanding the benefits of technology multiplied by 2.66 (1.11 to 1.75) in employees with a bachelor’s degree. The odds of recognizing that information is used by others multiplied by 5.05 (1.23 to 20.8) in employees who completed high school. Finally, the odds of agreement on desiring to learn more about the use of PD technologies at work multiplied by 0.16 (0.03 to 0.67) and 0.26 (0.07 to 0.96) in employees who completed elementary school or middle school, respectively.

English proficiency was also associated with participant perception of PD technology. When using “do not speak English” as reference category, the odds (95% CI) of agreement with being able to easily set up or back up the technology when not working multiplied by 2.99 (1.48 to 6.06), 4.55 (1.78 to 11.6), and 3.24 (1.19 to 8.84) when employees indicated intermediate, advanced, or native English proficiency, respectively. The odds of understanding the benefits of technology multiplied by 3.33 (1.45 to 7.69), by 4.54 (1.23 to 16.9), and by 6.17 (1.32 to 29.4) in employees with intermediate, advanced, and native English proficiency. Nonetheless, the odds of agreement in recognizing that employees get training when there is a new technology multiplied by 0.55 (0.32 to 0.95), 0.37 (0.18 to 0.74), and 0.12 (0.04 to 0.38) in workers with basic, intermediate, or native English proficiency. Finally, the odds of having a positive attitude toward new technology multiplied by 2.78 (1.18 to 6.62) for employees with intermediate English proficiency.

## DISCUSSION

Most research exploring the adoption of PD technologies has centered on the opinions reported by dairy producers and dairy management teams (Eastwood, 2008; Russell and Bewley, 2013; Eastwood et al., 2016), but less is known about the perception and challenges that hired labor faces on the implementation of precision technologies. In consequence, the aim of the current study was to explore in a convenience sample of dairies how employees would consider and adapt to the use of PD technologies in their daily work at the farm. Our approach was to complete this survey in a group of dairy operations that were under similar management and had a variety of PD technologies used by a large number of employees.

The demographics, education level, and English proficiency of the surveyed employees were of interest as potential factors affecting their perception and ability to effectively use PD technology. In specific, gender, age, and level of education have been identified as relevant variables associated with understanding and perception of the use of PD technology (Young, 2000; Bain and Rice, 2006). As foreign-born migrant workers represent a significant proportion of the labor force in U.S. agriculture (NORA, 2018), ethnicity and English proficiency were variables especially relevant. Notably, although previous studies have acknowledged the demographic profile of dairy farm employees in the United States, most of the reports are primarily limited to health and safety assessments at the workplace, without considering cultural challenges associated with specific tasks at the farm.

In accordance with findings in previous reports (Eastman et al., 2013; Rosecrance et al., 2013; Schenker and Gunderson, 2013; Menger et al., 2016; Martenies et al., 2020), the demographic profile of the respondent population resulted in more than 70% of employees who identified as male, 92.5% as Latino immigrants, and 93.2% as Spanish-speaking workers. The age range of the participants in this study was slightly younger compared to the dairy industry, with the most frequent interval being 21 to 30 yr, compared to the industry average of 34 yr (Mitchell et al., 2015). In addition, the level of education for participants in this study differed from other reports, with a significant number of employees who completed a bachelor’s degree (34.6%) and middle school (29.3%), compared to the previously reported 57% of dairy workers who completed up to elementary school (Eastman et al., 2013; Mitchell et al., 2015).

The current study did not identify significant associations between employees’ gender and responses related to their perception of PD technologies. These results are similar to those reported by a study in Canada that aimed to describe the adoption of eight specific PD technologies by the Canadian dairy industry (Jelinski et al., 2020). On the contrary, Groher et al. (2020) indicated that male respondents from Swiss ruminant operations were most likely to adopt PD technologies, compared to female respondents. These discrepancies across studies may be related to geographic and cultural differences, as well as the greater proportion of female respondents in the current research (23.9%), as compared to the Canadian study (1.9%) and the Swiss study (4%).

Previous research has identified an inverse association between the adoption of PD technology and age, where older dairy farmers are less likely to adopt new technologies within the operation (Hill et al., 2015; Groher et al., 2020; Shang et al., 2021). In the current research, age was initially associated with three of the perception and understanding of technology questions. However, only the use of technology to make decisions persisted when the associations were tested by logistic regression. The odds of agreeing with this statement were smaller in participants between 21 and 30 yr old, as compared with employees more than 50 yr old.

Contrary to previous studies on the use of PD technologies, our target was the dairy employee, who might have different interests and expectations when a new technology is implemented. Moreover, dairy employees have to adapt to a technology that the owner decides to adopt, so the perceptions within these two groups may significantly differ. The owner/operator has to consider the economical relevance of the investment by evaluating the cost, return on investment, maintenance, and other variables when considering a PD technology (Lovarelli et al., 2020). On the other hand, employees’ perceptions may be formed as a result of using a specific technology in their daily routine.

The current results showed that only 2.70% of the respondents feel uncomfortable (Likert scale scores 1 and 2 combined) when using technology in their daily routine, 1.60% did not understand how the technology works, and 6.54% reported that they did not receive training when there is a new technology. On the contrary, although centered on owner perceptions, Drewry et al. (2019) reported a significant level of discomfort with PD technologies (54.5%), a major lack of understanding (51.4%), and a notable absence of technology training (45.4%), resulting in barriers that inhibit technology adoption.

Regarding the magnitude and direction of the associations between the perception of PD technologies and demographic and general variables, both level of education and English proficiency seem to be relevant factors. A greater English proficiency was related to the ability to manage technologies that “are not working,” to the understanding of the benefits of technology, as well as to a positive attitude toward new technology. Interestingly, greater English proficiency was associated with smaller odds of recognizing that employees get training when there is a new technology. Similarly, higher education level was related to greater understanding of the benefits of technology and with recognizing that information is used by others. However, the willingness to learn more about the technologies used at the farm was lower in employees who completed elementary school or middle school compared with no formal education completed.

Some limitations should be recognized when interpreting the results of this study. First, the study was developed in a convenience sample of farms that were under the same ownership and general management, which is a significant limitation for the external validity of our findings. Second, comparisons with previous research are limited, given that most of the previous research has been focused on owner/producer perception. Third, the proportion of employees who completed a bachelor’s degree and middle school was high compared to values reported by other studies (Eastman et al., 2013; Mitchell et al., 2015). Fourth, as the respondents were asked to self-report their level of proficiency (i.e., language proficiency or their skills when using PD technology at work), responses might be affected by unrealistic respondent self-enhancement, which refers to situations in which people tend to rate their abilities as better than “average” (Kim et al., 2017). Finally, although a general definition of the term precision dairy farming was provided in the survey, the identification of precision technologies may vary across employees. Moreover, the specific technologies available for each employee are very variable depending on their area of work. Despite the identified limitations, the current study showed consistency with the general demographic profile for large dairies in the United States, although some differences in age and level of education were identified.

There is value in recognizing dairy employees’ emotions, skills, and personal and environmental limitations when using PD technologies, such as identifying opportunities to improve their adaptation to these technologies. The current research identified significant barriers described by dairy employees when using PD technologies, such as cold weather, high wind, not knowing the system’s main language, and vision limitations. Interestingly, in the current survey, visual impairment was identified as the second most frequent personal limitation in the use of this precision technology (24% of the respondents). Notably, issues in vision and eye care among dairy employees were recognized previously by Guifarro et al. (2019) and Rovai (2020). In the study by Guifarro et al. (2019), 20 out of 80 employees in four dairy farms in South Dakota needed further eye examination and 40% had never visited an eye care professional due to either cost or language barriers. Issues of color deficiencies were also detected and affected employees indicated that they usually make mistakes when using paint sticks for sorting cows for various protocols.

Also, the results showed that dairy employees associated positive emotions and feelings with the use of PD technology at work, and employees recognize practice, experience, and previous knowledge as personal skills that help them understand the technology. A significant proportion of PD technologies have been originally developed in Europe, where the intended final user is the farm owner. Moreover, in contrast with the U.S. dairy industry, these are smaller farms, with limited hired labor. In consequence, opportunities to improve efficient adaptation to technologies by a final user, different from the owner of the operation, should be explored by manufacturers. Offering more user-friendly systems that include language options may be worth considering.

## CONCLUSIONS

Overall, the dairy employees in the current study had a positive perception of PD technology, recognizing the benefits provided by its use in their daily work routine. The data from technologies were mostly considered a reliable source of information that can be used to make decisions. Education level and English proficiency were the main variables associated with the perception and understanding of these technologies. Moreover, challenges associated with language barriers and environmental limitations were recognized. Respondents indicated their desire to learn more about precision technologies implemented at work, which could eventually lead to improved efficiency at the dairy operation through innovations in the way users interact with these technologies, increasing employees’ motivation. These findings highlight some critical points affecting the success of the adaptation to precision technologies from the perspective of farm employees and could help enhance the efficient use of novel tools in livestock operations.

## Supplementary Material

txae036_suppl_Supplementary_Data
