# Supplementary material for: Employee perception of precision technology use at the dairy farm
Source: Transl Anim Sci. 2024 Mar 22;8:txae036. doi: 10.1093/tas/txae036 (PMC10983077; doi:10.1093/tas/txae036)
Supplement: txae036_suppl_Supplementary_Data [file txae036_suppl_supplementary_data.docx]

**Employee adaptation, perception and understanding of technology in the farm**

Objective 1: To assess how the farm employees adapt and recognize technologies implemented in dairy operations.

Objective 2: To identify possible challenges that farm employees face when using precision technologies in dairy operations.

**Demographic Questionnaire**

1. What gender do you identify as?

__ Male

__ Female
 __ Non-binary

__ Prefer not to answer

1. What is your age?

__ Under 20 years old

__ 21-30 years old

__ 31 to 40 years old

__ 41 to 50 years old

__ Over 51 years old

1. Which of the following best describes you?

__ Asian or Pacific Islander

__ Black of African American

__ Hispanic or Latino

__ Native American or Alaskan Native

__ White or Caucasian

__ Multiracial or biracial

__ A race/ ethnicity not listed here

1. What is the highest level of education you have achieved?

__ Middle School

__ High School

__ Bachelor's degree

__ Master's degree or above

__ Other

1. What is your employment status?

__ Full-time

__ Part-time

__ Contract/ Temporary

__ Internship

1. What is your primary language?

__ English

__ Spanish

__ Other _____________

1. What is your level of English?

__ Basic

__ Intermediate

__ Advanced

__ Native

__ Do not speak English

1. What is your level of Spanish?

__ Basic

__ Intermediate

__ Advanced

__ Native

__ Do not speak Spanish

1. Do you use a smartphone (iPhone, Samsung…)

__ Yes

__ No

1. Which department are you in?

__ Milking parlor

__ Animal caring: Hospital, trimming, maternity

__ Animal Reproduction: Breeding

__ Calf and heifer raising / caring

__ Farm office

__ Supervisor/ manager

__ Operator/ maintenance

__ Feeder

__ Administration

**Technology Questionnaire**

Precision Dairy Farming (PDF) is defined as the use of information and communication technologies to improve animal management.

**Technology Context**

1. Do you use any technology to do your work? (If yes, please answer question 2-4)

__ Yes

__ No

1. Do you recognize any technologie(s) at your work? (If answered no Q1)

__ Yes

__ No

1. How often do you use these technologies at work?

__ Every day

__ 2-3 times per week

__ 4-5 times per week

__ Twice per month

__ Once per month

1. Which of the following do you use at least once per week at work? (Mark all that apply)

__ PCDART Pocket

__ PCDART PC

__ TMR Software

__ CowManager

__ CowAlert

__ DelPro Animal tracking

__ DeLaval Brush

__ DeLaval Milking rotary/ line

__ Robot feed push

__ CattleEye

__ Lely Automatic Calf Feeder

__ Lely Robot milking

__ One Wand (RFID lector)

__ Sorting Gate (Milking Parlor)

__ I-Qube Pedometers

__ Lely Collars

__ Perfect Udder (pasteurization)

__ CASE/ John Deer tractor/ loader

__ Pivots

__ Plate reader (DM reading)

__ Scanner Code (Maintenance)

__ Ultrasound

__ Other ____________

1. What is the main purpose of the technology you frequently use? (mark all that apply)

__ Animal activity Monitoring

__ Animal Reproduction

__ Animal Health Status

__ Animal Nutrition Intake

__ Animal Production (Milk Yield)

__ Machinery maintenance and scanning

__ Pasture and Grazing Management

**Perception of Technologies**

Indicate your opinion for each of the following statements, based on the technology **you use the most** at work. If you do not use any technology to do your work, please select does not apply.

1. I feel comfortable using the technology in my daily routine.

__ Strongly Agree __ Somewhat agree __ Neither agree nor disagree__ Somewhat disgaree__ Strongly Disagree __ Does not apply

1. I understand how the technology works.

__ Strongly Agree __ Somewhat agree __ Neither agree nor disagree__ Somewhat disgaree__ Strongly Disagree __ Does not apply

1. I can easily set up or backup the technology if it is not working

__ Strongly Agree __ Somewhat agree __ Neither agree nor disagree__ Somewhat disgaree__ Strongly Disagree __ Does not apply

1. I feel comfortable with the language in the system I am using.

__ Strongly Agree __ Somewhat agree __ Neither agree nor disagree__ Somewhat disgaree__ Strongly Disagree __ Does not apply

1. I feel comfortable explaining to my colleague how to use the system.

__ Strongly Agree __ Somewhat agree __ Neither agree nor disagree__ Somewhat disgaree__ Strongly Disagree __ Does not apply

1. The technology help me to be more efficient in my daily operations

__ Strongly Agree __ Somewhat agree __ Neither agree nor disagree__ Somewhat disgaree__ Strongly Disagree __ Does not apply

1. I understand the benefits from the technology that I am using

__ Strongly Agree __ Agree __ Neutral__ Disagree __ Strongly Disagree __ Does not apply

1. I recognize that the information I enter in the system, someone else is using it

__ Strongly Agree __ Agree __ Neutral__ Disagree __ Strongly Disagree __ Does not apply

1. I feel confident to use the information from the technology that I am using

__ Strongly Agree __ Agree __ Neutral__ Disagree __ Strongly Disagree __ Does not apply

1. I use this technology or the information from the technology to make decisions in my job

__ Strongly Agree __ Agree __ Neutral__ Disagree __ Strongly Disagree __ Does not apply

1. The dairy provide me with all the resources I need to use the technology

__ Strongly Agree __ Agree __ Neutral__ Disagree __ Strongly Disagree __ Does not apply

1. The data resulting from monitoring systems is reliable.

__ Strongly Agree __ Agree __ Neutral__ Disagree __ Strongly Disagree __ Does not apply

1. I feel comfortable using a new technology when implemented at my work.

__ Strongly Agree __ Agree __ Neutral__ Disagree __ Strongly Disagree __ Does not apply

1. I get training when there is a new technology in my work.

__ Strongly Agree __ Agree __ Neutral__ Disagree __ Strongly Disagree __ Does not apply

1. I have positive attitude when adapting to a new technology at my work

__ Strongly Agree __ Agree __ Neutral__ Disagree __ Strongly Disagree __ Does not apply

1. I would like to learn more about using technologies at work

__ Strongly Agree __ Agree __ Neutral__ Disagree __ Strongly Disagree __ Does not apply

**Opportunities for technology adaptation**

1. Which of the following personal characteristics do you think might limit the use of technology? (Mark all that apply)

__ I have eye problems (nearsightedness, shortsightedness)

__ I have light sensibility

__ I do not know how to read

__ I do not know the main language of the technology

__ Other ____________________

1. Which of the following environmental characteristics do you think might limit the use of the technology? (Mark all that apply)

__ When it is too dark

__ When it is too bright

__ Extreme cold weather (below 20F)

__ Extreme hot weather (above 85F)

__ High wind

__ Other _________________

1. Which technology do you think will be beneficial for your work? (Beyond the technology that already exists in the dairy, describe the "ideal" technology that you think could facilitate your work)

____________________________________________________________________________________________________________________________________________________________________________________________________________________________________

1. In three words, describe how the technology implemented at work makes you feel?

__________________, __________________. __________________.

1. Mention three skills you consider that help you understand the technology at work.

____________________ , _______________________________ and ___________________.
